# Purified diet reduces intestinal IgA and alters the microbiota accordingly

**DOI:** 10.1017/S0007114525105916

**Published:** 2026-02-14

**Authors:** Masao Goto, Jun Watanabe, Motoi Tamura, Yuko Takano-Ishikawa, Manabu Wakagi

**Affiliations:** 1 Division of Food Function Research, Institute of Food Research, NARO, Tsukuba, Japan; 2 Obihiro University of Agriculture and Veterinary Medicine, Obihiro, Japan

**Keywords:** Purified diet, Intestinal IgA, T cell independent, Microbiota, Dysbiosis

## Abstract

Diet can affect health directly or by altering the gut microbiota; thus, there are strong interrelationships between the gut immune system, gut microbiota and diet. This study examined the effects of ingesting AIN-93M purified diet (PD) on gut immune function and gut microbiota in DO11·10 mice, in which T cell–dependent and T cell–independent (TI) IgA can be analysed separately. Ingestion of the PD for 2 weeks reduced both T cell–dependent and TI secretory IgA in the faeces compared with non-PD, whereas the diet did not affect T cell–dependent and TI serum IgA. Ingestion of the PD had no effect on systemic immune system splenocyte responses. Ingestion of the PD reduced intestinal tissue expression levels of B-cell activating factor and A proliferation–inducing ligand, cytokines involved in TI-IgA production and polymeric Ig receptor, which transports IgA into the intestinal lumen. Co-abundance group (CAG) analysis of the intestinal microbiota was conducted based on correlations between changes in the abundance of bacterial genera, and the correlations between CAG and IgA were determined. The *Allobaculum*-dominated CAG expanded following ingestion of the PD, accompanied by an inverse correlation with the decrease in faecal IgA, whereas the *Lactobacillus*-dominated CAG shrank relative to the *Allobaculum*-dominated CAG. These results suggest that TI-IgA suppresses the expansion of some intestinal bacteria and that ingestion of the PD induces dysbiosis via impaired IgA secretion into the intestinal lumen.

The small intestine not only absorbs nutrients, but it also functions as a lymphatic organ that responds to a variety of foreign substances, including the gut microbiota and food ingredients. Ingested foods are known to profoundly affect the gut microbiota^(1)^, and the gut microbiota in turn significantly impacts the health of the host. In particular, a strong relationship exists between the intestinal immune system and the gut microbiota^(2)^. When the intestinal immune system detects microbial metabolites, such as SCFA and microbial components, a variety of factors are secreted, such as antimicrobial peptides, inflammatory cytokines and IgA.

Large amounts of IgA are secreted into the mucosa by the intestinal immune system, and this IgA contributes to the maintenance of gut microbiota homeostasis. IgA not only eliminates or detoxifies invading pathogens and inhibits their growth, conversely, but it can also promote colonisation of the mucosa by commensal bacteria^(3–6)^. IgA can be classified into two types: T cell–dependent (TD)-IgA and T cell–independent (TI)-IgA. TD-IgA is produced by B cells stimulated by antigen-specific T cells^(7)^ and exerts both eliminating^(8,9)^ and inhibitory^(10)^ effects on pathogenic enterobacteria due to strong antigen specificity. By contrast, TI-IgA is produced by B cells stimulated by cytokines, such as B-cell activating factor (BAFF), which belongs to the TNF family, and A proliferation–inducing ligand (APRIL), which is secreted by dendritic cells and epithelial cells activated by microbial components, in the absence of cognate T cell help^(11)^. TI-IgA exhibits both poor antigen specificity and poly-reactivity against common intestinal bacterial molecules, such as lipopolysaccharide, flagellin and peptidoglycan^(8)^. Although the functions of TI-IgA remain to be fully elucidated, TI-IgA reportedly responds to changes within the commensal intestinal microbe population despite its antigen specificity^(12)^, and genetic mutations that hamper TI-IgA production are reportedly associated with increased susceptibility to inflammatory bowel disease^(13–15)^. These findings suggest that TI-IgA may stabilise the gut microbiota through poly-reactivity and contribute to maintaining health. On the other hand, the gut microbiota plays a pivotal role in the induction of both TI-IgA and TD-IgA production^(2,8,16,17)^. More recent studies have reported that bacterial metabolites such as SCFA^(18–20)^ and extracellular vesicles^(21)^ may affect IgA induction to a greater extent than taxonomic differences among bacteria^(16,17,22)^.

Recent studies have compared the health benefits of foods composed of diverse ingredients using non-purified diets (NPD) consisting of diverse natural ingredients with purified diets (PD) consisting of a few pure chemical products. PD feeding in experimental animals is reportedly associated with pregnancy rates^(23)^, infections and stress^(24)^ and depression^(25,26)^. Although not many reports have shown any involvement between PD and immune function, PD feeding in mice reportedly causes persistent candidiasis^(27)^, impairment of the intestinal mucus barrier and increased pathogen sensitivity^(28)^, decreased turnover of the small intestinal epithelium and decreased production of antimicrobial peptides^(29)^. Moreover, a recent report indicates that prolonged use of enteral nutrition supplements leads to dysbiosis in patients^(30)^. Enteral nutrition, which often consists of purified materials, is considered more beneficial for maintaining patient health than vascular nutrition. However, few studies have evaluated the effects of PD feeding on intestinal IgA production.

In this study, we examined the effect of PD ingestion on the immune response, focusing on intestinal IgA production using DO11·10 mice carrying a gene for a VDJ-reconstituted T cell receptor specific for chicken-egg ovalbumin (OVA) on a BALB/c background^(31)^. T cells that respond to non-OVA antigens are suppressed in DO11·10 mice because the introduced reconstituted OVA-specific T cell receptor gene suppresses the reconstitution of innate T cell receptor genes via allelic exclusion^(32,33)^. Therefore, any IgA observed in DO11·10 mice in the absence of OVA immunisation is presumed to be primarily TI-IgA. Furthermore, oral sensitisation of DO11·10 mice with OVA can induce OVA-specific TD-IgA production. We exploited this feature of DO11·10 mice to observe the effect of PD feeding on IgA production while distinguishing between TI-IgA and TD-IgA. In addition, changes in the gut microbiota following PD ingestion were also evaluated to elucidate the role of TI-IgA in maintaining homeostasis of the gut microbiota. Using this approach, we attempted to comprehensively analyse the effect of diet on IgA production and the gut microbiota.

## Experimental methods

### Animals

DO11·10 homozygous mice were purchased from the Jackson Laboratory and maintained in our specific pathogen-free animal facilities. Weaned animals were fed NMF (Oriental Yeast), a standard rodent NPD, and water *ad libitum* until the start of the experiments. The NMF diet is based on ingredients such as maize, wheat bran, defatted soyabean, defatted rice bran, alfalfa, fish meal, skim milk powder, soyabean oil and brewer’s yeast and designed to maintain good performance in breeding and for surgical experiments. Female mice aged 15–17 weeks were used in this study. Mice with abnormalities, including a weight reduction exceeding 20 % during the experimental period, were excluded from the experiments. All animal experiment protocols were reviewed and approved by the Animal Care and Use Committee of the National Agriculture and Food Research Organization, Japan (approval numbers H31-028 and 20B092FRI). Mice were kept on a 12-h light-dark (20.00–08.00 hours) cycle, with room temperature and humidity maintained throughout the year at 24 ± 1°C and 50 ± 5 %, respectively. Body weight was monitored at each exchange of diet and water, and mice were observed daily for signs of ill health. At the end of the experiment period, mice were euthanised by cervical dislocation and organs were harvested as required.

### Chemicals and reagents

OVA (fraction V grade) and Tris-buffered saline with 1 % bovine serum albumin were purchased from Sigma-Aldrich. OVA for oral sensitisation was purchased from Wako Pure Chemical Industries. TMB Microwell ELISA-peroxidase substrate was purchased from Surmodics (Eden Prairie). All other chemicals were of the highest purity available from commercial sources.

### Administration of experimental diets and sample collection

**Fig. 1.**
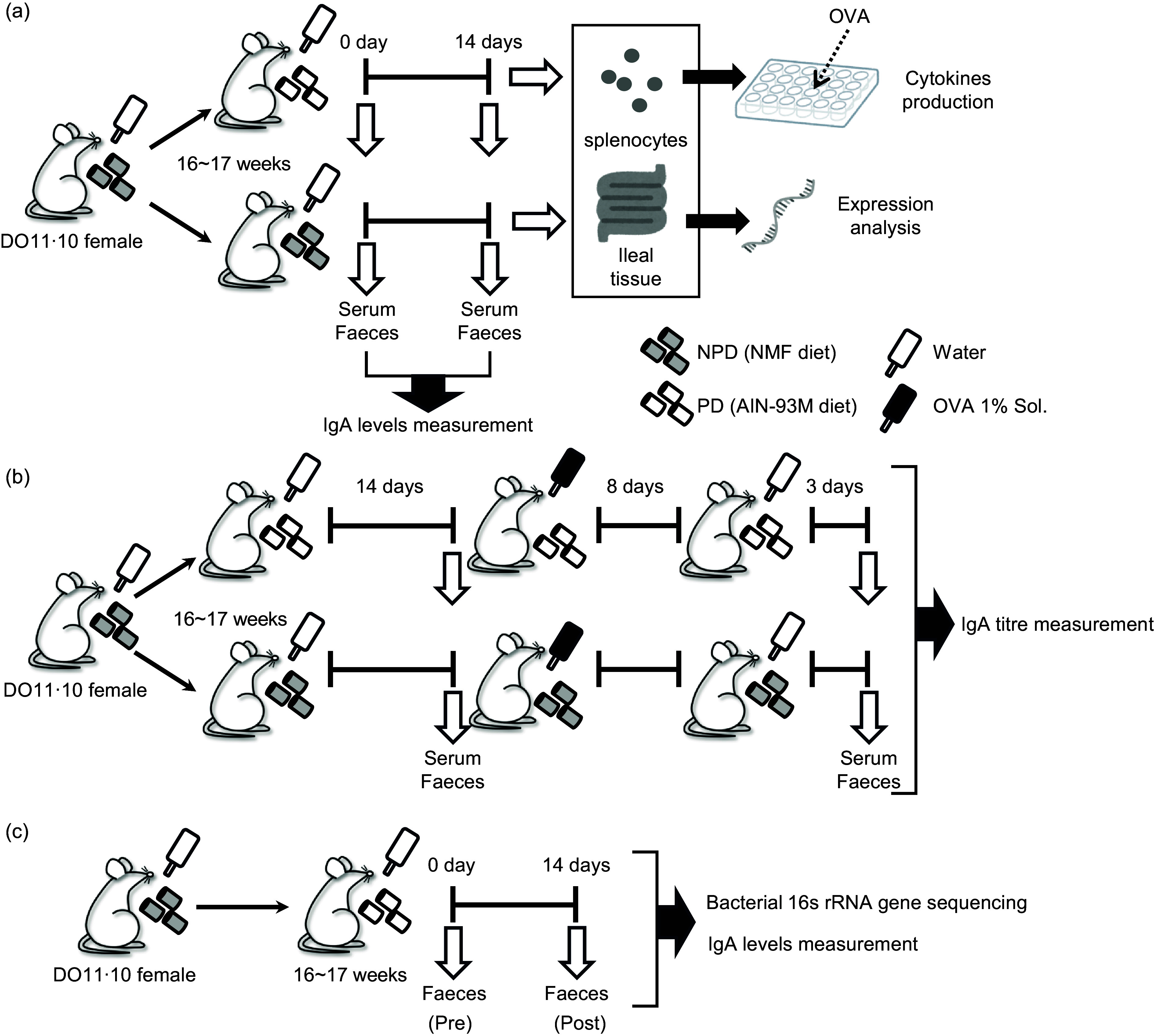
Experimental designs. Female DO11·10 mice were administered either non-purified diets (NPD) or purified diets (PD) for 2 weeks, and serum and faeces were collected before and after the administration to measure total IgA. Antigen-specific immune responses of splenocytes and gene expression analysis of ileal tissue were performed after administration of these diets (a). After 2 weeks of administration of the experimental diets, the mice were given 1 % (w/v) chicken-egg ovalbumin (OVA) solution as drinking water *ad libitum* for 8 d for oral sensitisation; after a 3-d antigen washout period, serum and faeces were collected, and antigen-specific IgA antibody titres were evaluated (b). Female DO11·10 mice were administered PD for 2 weeks, and faecal samples were collected before and after the administration for total IgA measurement and 16S rRNA gene-based gut microbiota analysis (c).

Mice aged 15–17 weeks were distributed randomly with 2–4 mice per cage and fed either NMF as NPD or AIN-93M as PD for 2 weeks *ad libitum* as the experimental diets. NMF and AIN-93M were purchased from Oriental Yeast Co., Ltd. AIN-93M is a standard PD for rodents derived from the National Institute of Nutrition (AIN) in 1993^(34)^ and contains no crude ingredients of animal or vegetable origin or dried yeast; the only carbohydrates are cellulose and maize starch. Faecal and serum samples were collected just before the start of experimental diet feeding and on experimental day 14 (Fig. 1(a)). These samples were collected between 13.00 and 14.00 and promptly stored at –30°C until further analysis. Faecal samples were individually placed into tubes provided with the BioMasher II kit (Fujifilm Wako Pure Chemicals) and weighed, with approximately 100 mg obtained from a single sampling per subject.

### Oral antigen sensitisation and sample collection

After 2 weeks of experimental diet administration, the mice were given 1 % (w/v) aqueous OVA solution for 8 d, followed by water for 3 d *ad libitum*. During sensitisation, the mice were continuously fed the same diet. Faecal and serum samples were collected as described above, just before the oral antigen administration and on day 11 after the initiation of antigen administration (Fig. 1(b)).

### Measurement of antigen-specific cytokine production following stimulation of immune cells with *ex vivo* antigen

After 2 weeks of experimental diet administration, the mice were euthanised via cervical dislocation, and splenocytes were prepared using a standard method. Splenocytes were cultured in RPMI-1640 medium (Sigma-Aldrich) containing 2-mercaptoethanol (50 µM), penicillin (100 U/ml), streptomycin (100 µg/ml) and 10 % foetal calf serum (PAA Laboratories) in culture plates (Nunc).

To measure levels of cytokines in culture supernatants, splenocytes were cultured at 3 × 10^5^ cells/well in a total volume of 300 µl per well and stimulated with OVA (7·5 µM). Cells were cultured in duplicate wells of 96-well plates. Culture supernatants were collected at 48 h (for IL-2, IL-4 and IL-5 analysis) and 72 h (for IL-6, IL-10, IL-12 and IFN-*γ* analysis) after OVA stimulation. Supernatants were stored at –30°C until analysis. Cytokine levels were measured using Mouse Cytokine ELISA Ready-SET-Go! kits (eBioscience) according to the manufacturer’s instructions.

### Analysis of A proliferation–inducing ligand, B-cell activating factor and polymeric Ig receptor expression in ileal samples

We collected 3 cm of ileal tissue from the ileocecal valve. Sections were prepared and permeabilised using RNAlater (Qiagen) and then stored at –30°C until analysis. Total RNA was extracted from the samples using an RNeasy Mini kit (Qiagen KK), according to the manufacturer’s instructions. Total RNA (1 µg) was then reverse-transcribed into cDNA using SuperScript II RT and random primer oligonucleotides (Takara Bio) in a 20-µl final volume.

Quantitative RT-PCR was performed using a Real-Time QPCR System (QuantStudio; Thermo Fisher Scientific Inc.) in a total volume of 20 µl in the presence of cDNA (1 µl solution) plus 19 µl of reaction mixture (0·4 µl each of 10 µM primer and 10 µl of 2 × KAPA SYBR FAST qPCR Master Mix Universal (Kapa Biosystems Inc.)). Quantitative PCR was carried out under the following conditions: one cycle of 3 min at 95°C, 40 cycles of 3 s at 95°C and 30 s at 62°C for analysis of the expression of *April*, *Baff* and *pIgR*. The following primer sets were used: April-F: 5’-ACCCAGAAGCACAAGAAGA AGC-3’; April-R: 5’- GTACTGGTTGCCACATCACCTC-3’; Baff-F: 5’-TACACATTTGTTCCATGGCTTC-3’; Baff-R: 5’-GCAAGCTGA ATCTCATCTCCTT-3’; pIgR-F: 5’-GCTCCAAAGTGCTGTTCTC C-3’; pIgR-R: 5’-TTGCTGTGTGTCTGGAGAGG-3’; Gapdh-F: 5’-ATCCCAGAGCTGAACG-3’; Gapdh-R: 5’-GAAGTCGCAGGAG ACA-3’.

The relative amount of each *April*, *Baff* and *pIgR* transcript was normalised to the amount of *Gapdh* transcripts in the same cDNA sample. To ensure the specificity of the PCR method, melting curve analysis was conducted after amplification. The melting curves were obtained by heating samples at temperatures from 60°C to 95°C with continuous fluorescence monitoring.

### Faecal DNA extraction and 16S rRNA gene sequencing

DNA was extracted from faeces using a ZircoPrep Mini kit (Nippon Genetics) and a QIAamp DNA Stool Mini kit (Qiagen). DNA samples (10 ng each) were used as templates to amplify the V3 and V4 regions of the 16S rRNA gene by PCR using the 341F (5’-CCTACGGGNGGCWGCAG-3’) and 806R (5’-GGACTACHVGGGTWTCTAAT-3’) primers joined to Illumina overhang adaptor sequences^(35)^. A second PCR was carried out to add barcodes to each sample using a Nextera XT Index kit v2 (Illumina). After quantification, amplicons were pooled in equal amounts, and pair-end 2 × 300 bp sequencing was performed using a MiSeq system (Illumina) and MiSeq Reagent kit v3 (Illumina).

### Metataxonomic analysis

Sequences, in demultiplexed format, were analysed using QIIME2 2023·2 (https://qiime2.org). Merged pair-end reads were filtered by length and quality, and DADA2^(36)^ was used to identify the amplicon sequence variants. Amplicon sequence variants assigned as originating from chloroplasts and mitochondria were eliminated from further analyses. The GreenGenes database (ver 13_8, https://doi.org/10.1038/ismej.2011.139) was used to annotate the taxonomic information for representative sequences of amplicon sequence variants. Alpha-diversity was estimated according to Chao-1, Shannon index, Simpson index and Simpson index of evenness, whereas beta-diversity was evaluated using weighted UniFrac distances by rarefying the feature table at a consistent sampling depth of 40 000.

All genus-like-level groups were used for bacterial co-abundance group (CAG) analyses to highlight common patterns of abundance among genera^(37)^. The co-abundance between each pair of genera was evaluated by calculating the Kendall correlation coefficient and displayed in a heatmap, and the genera were hierarchically clustered based on Pearson’s correlation metric and average linkage in R software, version 4.3.1. The transition type of each CAG by the dietary intervention indicated the sum of Z-scores calculated from the relative abundance of genera classified in the same CAG. Network plots depicting correlative relationships among the genera were generated using Cytoscape, version 3.10.1^(38)^.

### Measurement of IgA levels in serum and faecal extracts

Faecal samples for IgA measurement were suspended in Tris-buffered saline with 1 % bovine serum albumin (100 mg of faeces per millilitre), homogenised at room temperature for 1 min using a BioMasher II and a Powermasher II according to the manufacturer’s instructions and centrifuged at 7700 *g* for 5 min at 4°C. IgA levels in serum and faecal extracts were measured using a Mouse IgA ELISA Quantitation Set (Bethyl), according to the manufacturer’s instructions. The titre of OVA-specific IgA was measured using a sandwich ELISA, as follows. Maxisorp plates (NUNC) were coated by incubation with OVA in PBS (100 µg/ml) at 4°C overnight. The plates were then washed with wash buffer (PBS containing 0·05 % Tween 20) and blocked with Tris-buffered saline with 1 % bovine serum albumin (Sigma-Aldrich). After washing, samples were diluted with blocking buffer, added to the plate wells and incubated at 4°C overnight. The plates were then washed with wash buffer, followed by the addition of horseradish peroxidase–labelled anti-mouse IgA (Bethyl) and incubation for 1 h at room temperature. TMB substrate was then added to each well, and the absorbance was measured (450 nm) after stopping the reaction. An IgA titre standard curve was prepared by stepwise dilution of serum samples from DO11·10 mice orally administered OVA (titre set at 4800 KU), which were prepared separately from this study. OVA levels in faecal samples used for OVA-specific IgA measurements were confirmed to be below the detection limit using an OVA ELISA kit (ITAEA).

### Statistical analysis

Since this is the first study to analyse the effects of PD on TI-IgA and TD-IgA and gut microbiota in DO11·10 mice, no data on effect size were available for sample size calculation or minimally detectable effect sizes. Based on a study^(28)^ analysing the effects of a reduced fibre diet on the colonic barrier function of gnotobiotic mice in groups of 6–30 mice per group, the present study used fifty-four mice each on purified and not-purified diets for all experiments.

**Fig. 2.**
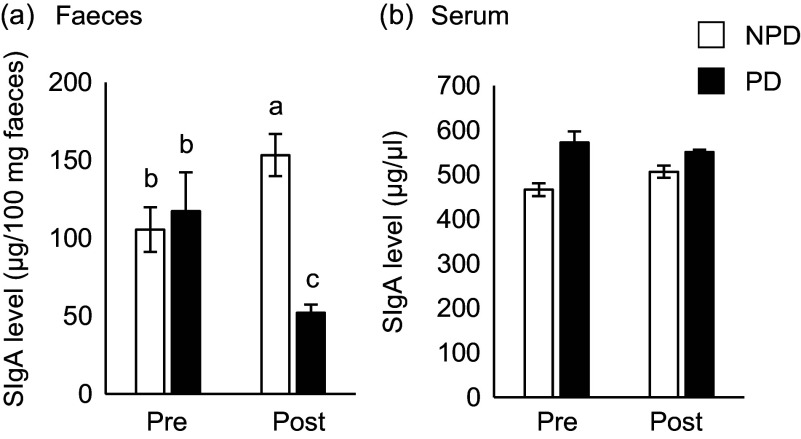
Effects of purified diet (PD) ingestion on the T cell–independent-IgA in faeces and sera. DO11·10 mice were fed either with non-purified diets (NPD) or PD for 2 weeks. Faecal and serum samples were collected before (pre) and after (post) the experimental feedings. NPD and PD groups are shown in open columns and closed columns, respectively. Total IgA in the faecal (a) and serum (b) samples was quantified by ELISA. Data shown are expressed as mean (se) ((a): *n* 34, (b): *n* 11), representing the cumulative number of mice used across six independent experiments for (a) and two independent experiments for (b). Values without the same letter differ significantly (*P* < 0·05, Steel–Dwass multiple comparison test).

**Fig. 3.**
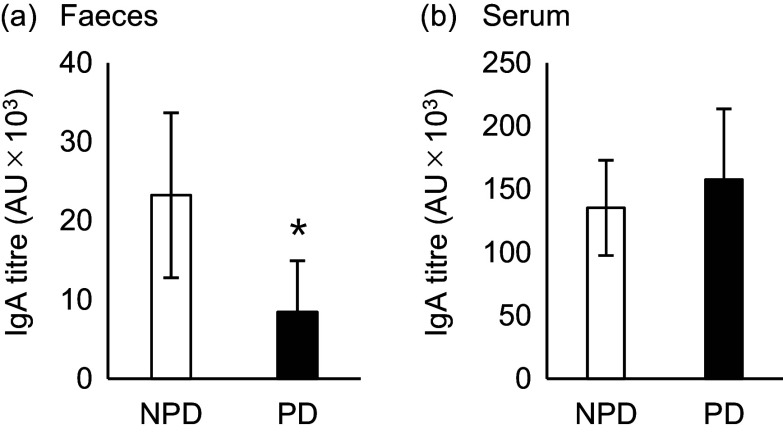
Effects of purified diet (PD) ingestion on the T cell–dependent-IgA in faeces and sera. DO11·10 mice were fed either with non-purified diets (NPD) or PD for 2 weeks and were sensitised by oral administration of chicken-egg ovalbumin (OVA) for 8 d. Titres of OVA-specific IgA in the faecal (a) and serum (b) samples were quantified by ELISA. NPD and PD groups are shown in open columns and closed columns, respectively. Data shown are expressed as mean (se) (*n* 8) (*P* < 0·05, Mann–Whitney *U* test).

**Fig. 4.**
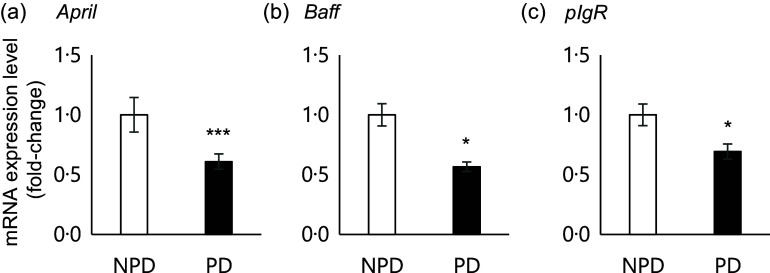
Effects of purified diet (PD) ingestion on the expression levels of T cell–independent (TI)-SIgA-related genes. DO11·10 mice were fed either with non-purified diets (NPD) or PD for 2 weeks, and the expression of genes related to TI-SIgA in the ileal tissue was analysed. *April*, A proliferation-inducing ligand (a); *Baff*, B-cell activating factor belonging to the TNF family (b); *pIg*R, polymeric Ig receptor (c). Total RNA was extracted from ileal tissue, and expression levels were evaluated by RT-PCR. Expression levels were normalised to the amount of *Gapdh* in the same cDNA. NPD and PD groups are shown in open columns and closed columns, respectively. Data shown are expressed as mean (se) (*n* 10), representing the cumulative number of mice used across two independent experiments (*P < 0·05, ***P < 0·001, Mann–Whitney *U* test).

Statistical comparisons of non-gut microbiota data were performed using the Steel–Dwass multiple comparison tests (Fig. 2) and Mann–Whitney *U* test (Figs. 3 and 4 and Table 1). Differences showing *P* < 0·05 were considered statistically significant. All statistical analyses were performed using EZR 1.54 software (Saitama Medical Center, Jichi Medical University), which is a graphical user interface for R (The R Foundation for Statistical Computing).

**Table 1.** Effects of PD ingestion on the antigen-specific responses of systemic immune cells

(pg/ml)	PD		NPD	
	Mean	se	Mean	se
IL-2	1644	146	1529	103
IL-4	91·1	26·6	55·3	10·4
IL-5	61·6	30·8	31·9	6·2
IL-6	414·9	69·7	362·0	25·3
IL-10	2764	397	2462	352
IL-12	54·7	3·6	57·5	4·8
INF-*γ*	19 214	2426	19 987	2585

PD, purified diets; NPD, non-purified diets.

DO11.10 mice were fed either with PD or NPD for 2 weeks, and their splenocytes were stimulated with 7·5 μM chicken-egg ovalbumin. Cytokines in the culture supernatant were quantified by ELISA. Data shown are expressed as mean (se) (*n* 16).

## Results

### Purified diet ingestion reduces intestinal T cell–independent-IgA secretion without affecting serum T cell–independent-IgA levels

To elucidate the effect of PD ingestion on intestinal and serum IgA levels, we measured total IgA in serum and faeces samples from mice fed PD or NPD *ad libitum* for 2 weeks before and after ingestion (Fig. 1(a)). As shown in Fig. 2(a), faecal total IgA levels were significantly reduced after 2 weeks of PD ingestion (*P* = 0·014), although the levels were significantly increased by ingestion of NPD (*P* = 0·032). By contrast, no significant differences in serum total IgA levels due to the dietary intervention were observed (Fig. 2(b)). In addition, no significant differences in food and water intake or body weight gain were observed between the groups (online Supplementary Table 1).

### Purified diet ingestion reduced intestinal T cell–dependent-IgA secretion without affecting serum T cell–dependent-IgA titre

Next, we evaluated the effect of diet on TD-IgA secretion. OVA-specific IgA was induced by oral OVA sensitisation 2 weeks after NPD *v*. PD ingestion, and titres of both faecal and serum IgA were evaluated (Fig. 1(b)). The faecal IgA titre was significantly lower in the PD group than the NPD group (Fig. 3(a), *P* = 0·028). By contrast, no significant difference in serum IgA titre was observed between the NPD and PD groups (Fig. 3(b)).

### Purified diet ingestion did not alter the antigen-specific responsiveness of systemic immune cells

Previous research suggested that PD ingestion reduces the secretion of TD-IgA into the intestine without affecting the production of TD-IgA. Therefore, an *ex vivo* assay using splenocytes from mice of the PD and NPD groups was used to confirm that PD ingestion does not affect antigen-specific systemic immune responses regulated by T cells. The spleen is an organ of the systemic immune system, and splenocytes consist of various subpopulations of immune cells, such as T and B cells, dendritic cells, NK cells, macrophages and monocytes. As such, splenocytes are commonly used in *ex vivo* assays to assess the antigen-specific responsiveness of systemic immune cells. We evaluated the levels of various cytokines (IL-2, -4, -5, -6, -10, -12 and IFN-*γ*) in culture supernatants following antigen stimulation (Table 1). No significant differences in the levels of these cytokines were observed between the NPD and PD groups.

### Purified diet ingestion downregulates ileal expression of T cell–independent-IgA production– and IgA transport–related genes

Previous results suggested that PD ingestion does not affect TD immune responses of systemic immune cells. As we found that PD ingestion reduced faecal total IgA levels without affecting serum total IgA (Fig. 2), we examined the expression of genes related to TI-IgA production in ileal tissue. In the PD group, significant downregulation of several genes was observed, including *April* (*P* = 0·0001) and *Baff* (*P* = 0·036), which encode cytokines that induce TI-IgA class switching in B cells, and *pIgR*, which encodes a protein that binds to IgA to mediate secretion into the lumen via transcytosis (Fig. 4).

### Purified diet ingestion causes dysbiosis of the intestinal microbiota

**Fig. 5.**
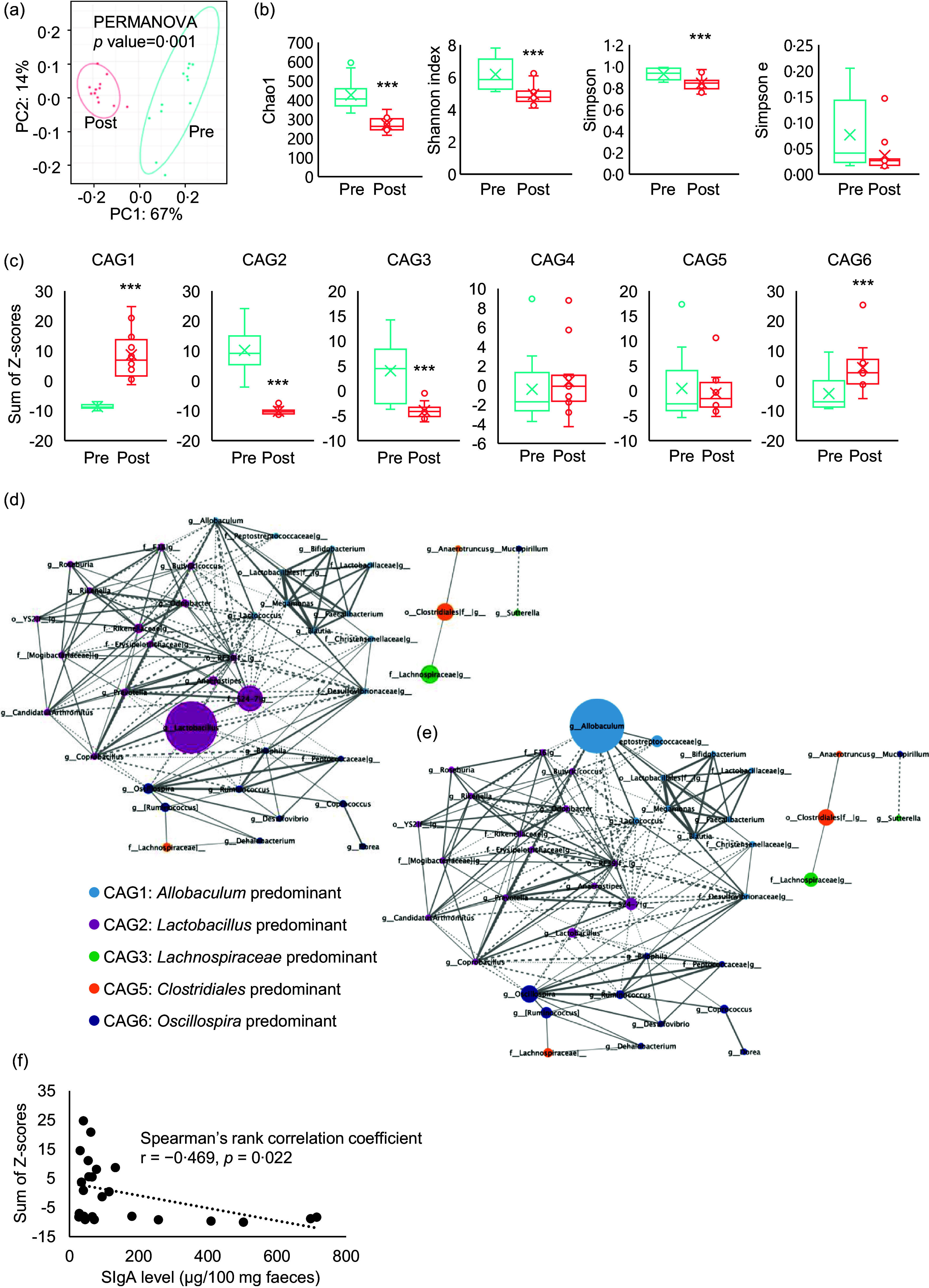
Effects of purified diet (PD) ingestion on the composition, structure, and IgA-related features of faecal microbiota. Principal coordinate analysis (PCoA) based on weighted UniFrac distances (a) and *α*-diversity parameters Chao, Shannon index, Simpson index and Simpson index of evenness (b) of faecal microbiota of mice before (pre) and after (post) the ingestion of PD for 2 weeks (*n* 12) (**P < 0·01, ***P < 0·001, Kruskal–Wallis pairwise test). Sum of Z-scores converted from relative abundance of genera belonging to co-abundance group (CAG) 1–6 (c) of faecal microbiota of mice before (Pre) and after (Post) the ingestion of PD for 2 weeks (*n* 12) (***P < 0·001, Wilcoxon signed rank test). Network plot highlighting relationships between genera in the CAG of faecal microbiota of mice before and after the ingestion of PD. Correlations among genera in the CAG of the faecal microbiota before (d) and after (e) the ingestion of PD are shown as network plots. The colours and circle size indicate the 6 CAG and genus abundance, respectively. Dotted and solid lines show significant positive and negative correlations (*P* < 0·05, Spearman’s correlation test) between the genera with an absolute coefficient value greater than 0·7, and the width of lines indicates absolute coefficient values. Correlation of faecal total IgA levels and relative abundance of genera belonging to CAG1 (f). Faecal IgA levels and the sum of Z-scores of relative abundances of genera in CAG1 of mice both before and after the ingestion of PD for 2 weeks were plotted. Spearman’s rank correlation test was conducted for the correlation analysis (*P* = 0·022).

The faecal microbiota was compared before and after PD ingestion (Fig. 1(c)). PD ingestion clearly altered the composition of the faecal microbiota (Fig. 5(a)), and all *α*-diversity parameters of the microbiota exhibited decreasing trends after PD ingestion (Chao1: *P* < 0·0001, Shannon index: *P* = 0·0015, Simpson index: *P* = 0·0047, Simpson index of evenness : *P* = 0·083) (Fig. 5(b)).

Analysis of correlations between changes in the abundance of each genus due to PD ingestion resulted in the classification of six CAG, among which *Allobaculum*, *Lactobacillus*, *Lachnospiraceae*, *Bacteroides*, *Clostridiales* and *Oscillospira* were the predominant bacterial genera (online Supplementary Fig. 1). Evaluation of the sum of genus abundance Z-scores in each CAG for each individual mouse revealed that PD ingestion significantly increased *Allobaculum*-predominant CAG1 (*P* < 0·001) and *Oscillospira*-predominant CAG6 (*P* < 0·0092), but particularly CAG1. By contrast, PD ingestion significantly reduced *Lactobacillus*-predominant CAG2 (*P* < 0·001) and *Lachnospiraceae*-predominant CAG3 (*P* < 0·001), whereas *Bacteroides*-predominant CAG4 and *Clostridiales*-predominant CAG5 were not significantly affected by PD ingestion (Fig. 5(c)). Network analysis of the correlations between CAG revealed a weak negative correlation between CAG1 and CAG2 (Fig. 5(d) and (e)).

### Increases in *Allobaculum*-dominated co-abundance group inversely correlated with the decrease in faecal IgA caused by purified diet ingestion

We hypothesised that PD ingestion suppresses the activity of non-T immune cells in the intestine, with resulting decreases in the production of TI-IgA in the intestine. In addition, CAG analysis revealed that PD ingestion affected the transition of CAG. Hence, to examine the relationship between transitions of CAG and the PD-induced decrease in intestinal TI-IgA, we examined the correlation between the sum of Z-scores of the abundance of each genus within a CAG and the level of faecal IgA. The results showed an inverse correlation between CAG1 and IgA level (Fig. 5(f)), suggesting that TI-IgA suppressed the expansion of bacteria belonging to CAG1.

## Discussion

PD ingestion reportedly affects gastrointestinal homeostasis. Several studies have examined the effect of PD ingestion on intestinal barrier function^(27–29)^. In this study, we used AIN-93M as the PD. AIN-93M is richer in several nutrients than the NPD, including vitamin A, and it is designed to have no effect on growth, pregnancy or lactation in mice during the first year of life^(34,39)^. To clarify the effect of PD feeding on the production of TI- and TD-IgA, we used DO11·10 mice, in which IgA is predominantly TI-IgA in the absence of OVA sensitisation. In this study, ingestion of PD for 2 weeks significantly reduced faecal total IgA levels in DO11·10 mice (Fig. 2(a)). Furthermore, PD ingestion significantly suppressed the elevation in faecal TD-IgA titre induced by oral OVA sensitisation, as with TI-IgA (Fig. 3(a)). By contrast, there were no significant differences in body weight gain between the PD and NPD groups, and there were no significant differences in serum TI- and TD-IgA and antigen-specific immune responses of splenocytes, which are part of the systemic immune system (Figs. 2(b) and 3(b), Table 1). Thus, these data strongly suggest that the decrease in faecal IgA due to PD ingestion is not caused by suppression of the systemic immune response but rather by impairment of the intestinal immune response.

In intestinal tissues, ingestion of PD for 2 weeks significantly reduced the expression levels of *April*, *Baff* and *pIgR* (Fig. 4). pIgR is secreted by epithelial cells and plays an essential role in the secretion of IgA into the intestinal lumen^(40)^. APRIL and BAFF are cytokines produced by intestinal epithelial cells or plasmacytoid dendritic cells in the lamina propria^(41)^, and they act directly on B cells to induce TI-IgA production^(42)^. By contrast, APRIL and BAFF are not associated with the induction of TD-IgA^(42)^. Thus, PD ingestion would not be expected to affect TD-IgA induced by oral antigen sensitisation. Indeed, PD ingestion did not affect serum TD-IgA levels (Fig. 3(b)) but instead reduced faecal TD-IgA as well as TI-IgA levels (Fig. 3(a)). These results suggest that the major mechanism by which PD ingestion reduces faecal TI-IgA and TD-IgA levels involves downregulation of pIgR expression in the intestinal tissues. Expression of pIgR is regulated by a complex mechanism that involves both RelA and RelB^(43)^. Secretory leukocyte protease inhibitor downregulates pIgR expression through the NF-κB signalling pathway by inhibiting degradation of IκB*β* ^(44)^. Maria *et al*.^(40)^ reported that bacterial stimuli induce pIgR expression in HT-29 cells via the NF-κB signalling pathway. Kento *et al*.^(45)^ reported that ingesting high amounts of *β*-glucan barley alters the intestinal microbiota and upregulates pIgR expression. Based on our findings and these previous reports, we speculate that PD lacks certain nutritional components such as grains and other raw materials, leading to the downregulation of pIgR expression. While our analysis was conducted at the mRNA level, further investigation would be valuable to confirm these observations, including at the assessments at the protein level.

Food-derived innate immune receptor agonists, as well as intestinal bacterial compounds and metabolites, can stimulate the intestinal immune system. Most intestinal commensal bacteria can induce intestinal TI-IgA production^(8)^, and these inducers can be broadly categorised as either the bacteria themselves, such as segmented filamentous bacteria (SFB)^(46)^, or secretions such as soluble proteins from *Lactobacillus rhamnosus* GG^(47)^, SCFA metabolised from dietary fibre^(19)^ and extracellular vesicles secreted by commensal bacteria that assimilate proteins in foods^(21)^. Ingested foods are known to affect both the composition and metabolism of the bacteria that make up the intestinal microbiota^(48)^. The assimilation of a diverse range of complex components also reportedly increases the metabolic efficiency of the intestinal microbiota, and the resulting metabolites contribute to improving host health^(28,49)^. The raw materials used in NPD, such as wheat bran, soyabean, rice bran and brewer’s yeast, are rich in dietary fibre^(50,51)^, which may be metabolised by intestinal bacteria and thereby promote IgA production. The composition of the PD examined in this study was apparently less diverse than that of the NPD. Indeed, PD feeding was shown to decrease the abundance of SFB^(52)^ or induce dysbiosis by reducing the diversity of the gut microbiota^(24)^. In our study, ingestion of PD for 2 weeks significantly reduced the *α*-diversity parameters of the gut microbiota and significantly altered the beta-diversity (Fig. 5(a) and (b)). The diverse components of NPD provide various bacterial species with means of survival, thereby contributing to the formation of complex inter-bacterial relationships. In contrast, the relatively simple nutritional environment provided by PD is thought to favour a limited number of bacterial species.

It is now believed that there is essentially no relationship between the taxonomic position of bacteria and IgA production^(17,22)^; rather, bacterial activity and metabolism exert a major effect on IgA production. Therefore, to clarify patterns in the microbiota, we classified the intestinal bacterial genera into CAG based on co-abundance associations before and after PD feeding for 2 weeks. Each resulting CAG included bacteria of different families (online Supplementary Fig. 1), and PD feeding significantly increased the abundance of CAG1 and CAG6 and significantly decreased that of CAG2 and CAG3 (Fig. 5(c)). *Allobaculum* and *Oscillospira*, considered potential causative agents of inflammatory bowel disease, were the most abundant bacteria in CAG1 and CAG6, respectively^(4,53)^. By contrast, *Lactobacillus*, the most abundant bacteria in CAG2, secretes lactic acid and soluble proteins that induce IgA production^(47)^, whereas *Lachnospira* and *Roseburia*, the major CAG3 genera, secrete acetic acid^(18)^ and lactic acid^(19)^, respectively, and are largely responsible for inducing IgA production^(54,55)^. These results suggest that ingestion of PD decreases the abundance of bacteria that promote IgA production and increases the abundance of enteritis-causing bacteria. As such, we analysed the correlation between the sum of the Z-scores of the relative abundances of the genera classified into each CAG and the faecal IgA level. We found a significant inverse correlation between CAG1 and faecal IgA level (Fig. 5(f)).

TI-IgA binds to diverse intestinal commensal bacteria across multiple species^(56)^ and ‘coats’ the surface of the bacteria^(8,57)^ to maintain homeostasis of the intestinal microbiota^(4–6)^. The density of this coating depends on the affinity of the IgA for the bacteria. Commensal bacteria that cause opportunistic infections are generally heavily coated with IgA, which inhibits their growth^(58)^ and motility^(59)^. SFB are also heavily coated with IgA, and decreased IgA production recovers the proliferation of SFB^(60)^. Furthermore, similar to SFB, IgA also heavily coats species of *Allobaculum*, the most abundant bacterial genus in CAG1^(4)^. These data thus suggest that the proliferation of bacteria belonging to CAG1 may be inhibited by IgA on the cell surface, and this inhibition may be alleviated by decreasing the production of IgA through feeding of PD, resulting in an expansion of CAG1 bacteria. As CAG1, the abundance of genera in CAG6 increased significantly following PD feeding (Fig. 5(c)), but no correlation with IgA level was observed. *Oscillospira*, the most abundant CAG6 bacteria, is reportedly coated with IgA more lightly than SFB and *Allobaculum* ^(4,61)^, which may explain the lack of detectable correlation between CAG6. IgA level was not correlated with either CAG2 or CAG3, even though *Lactobacillus* ^(57,62,63)^ and *Lachnospiraceae* ^(64)^ reportedly induce IgA production. Bacteria that induce IgA production, such as *Lactobacillus*, are less coated with TI-IgA, facilitating their colonisation of the gut^(4,8,65–67)^. This might suggest that the reduction in IgA level decelerates intestinal colonisation by bacteria within these CAG, leading to a further reduction in IgA level. In this case, IgA level and bacterial abundance likely exhibit a nonlinear relationship.

Dietary components may also play a role in IgA induction. These dietary factors may have obscured the correlation between IgA and gut bacteria that regulate IgA production. Intestinal bacteria also affect each other’s proliferation^(68)^. The network analysis conducted in this study also suggested that a weak antagonistic relationship exists between CAG1 and CAG2 (Fig. 5(d) and (e)). These results also suggest an association between the observed expansion of CAG1 and decreases in both IgA level and CAG2 abundance. On the other hand, our interpretation was based on findings from previous studies evaluating IgA-coating intensity of the murine gut microbiota. Additionally, it should also be noted that IgA-coated bacteria were neither isolated nor sequenced in this study. Therefore, IgA-coated bacteria should be analysed in future studies to validate these speculative interpretations and enhance biological relevance.

PD lacks a wide variety of naturally derived components, and the changes observed in intestinal IgA and microbiota composition induced by PD intake are strongly suggested to be associated with the deficiency of these components. Although it is currently difficult to identify the responsible components, it is possible that components directly inducing IgA production may be present in NPD. For example, *β*-sitosterol, a phytochemical in soyabeans and maize included in NPD, has been reported to promote SIgA production in the ileum^(69)^. *β*-glucans, which are known to induce pIgR expression in intestinal epithelial cells^(44)^, are also abundant in yeast^(70)^ and cereals^(71)^. Alternatively, dietary components may alter immune responses via modulating microbiota activities. In this study, PD intake reduced CAG2 and CAG3, which include SCFA-producing bacteria (Fig. 5(c)). SCFA, which promote IgA production, are generated by microbial fermentation of dietary fibres, and wheat bran and rice bran are rich in such fibres^(50)^. On the other hand, various flavonoids have been reported to possess antimicrobial properties^(72)^. A typical example of antimicrobial flavonoids is isoflavones, which are abundant in soyabeans^(73)^. Their absence in PD may have allowed the expansion of some bacterial populations. Furthermore, the existence of gut bacteria that suppress IgA production to evade host immunity was reported recently^(74)^, and it may be possible that the increase of such bacteria contributed to the reduction in IgA levels.

In this study, we showed that ingestion of PD for 2 weeks reduced faecal IgA production via modulation of local gene expression in intestinal tissues without affecting the systemic immune system, and that the resulting PD-associated decrease in faecal TI-IgA may cause dysbiosis. Recent reports indicate that prolonged use of enteral nutrition supplements leads to dysbiosis that is difficult to reverse by administration of probiotics^(30)^. If IgA is involved in maintaining homeostasis of the intestinal microbiota but is reduced by long-term administration of enteral nutrition, the ability of supplemented probiotic strains to colonise the gut would likely be impaired. Further studies are needed to more clearly elucidate the role of IgA in regulating the intestinal microbiota.

## Supporting information

Goto et al. supplementary material 1 Goto et al. supplementary material

Goto et al. supplementary material 2 Goto et al. supplementary material

## Supplementary material

For supplementary material/s referred to in this article, please visit https://doi.org/10.1017/S0007114525105916.
