# Supplementary figures and images for: Purified diet reduces intestinal IgA and alters the microbiota accordingly
Source: Br J Nutr. 2025 Dec 17;135(3):276–85. doi: 10.1017/S0007114525105916 (PMC12912838; doi:10.1017/S0007114525105916)

Height

0 10 20 30

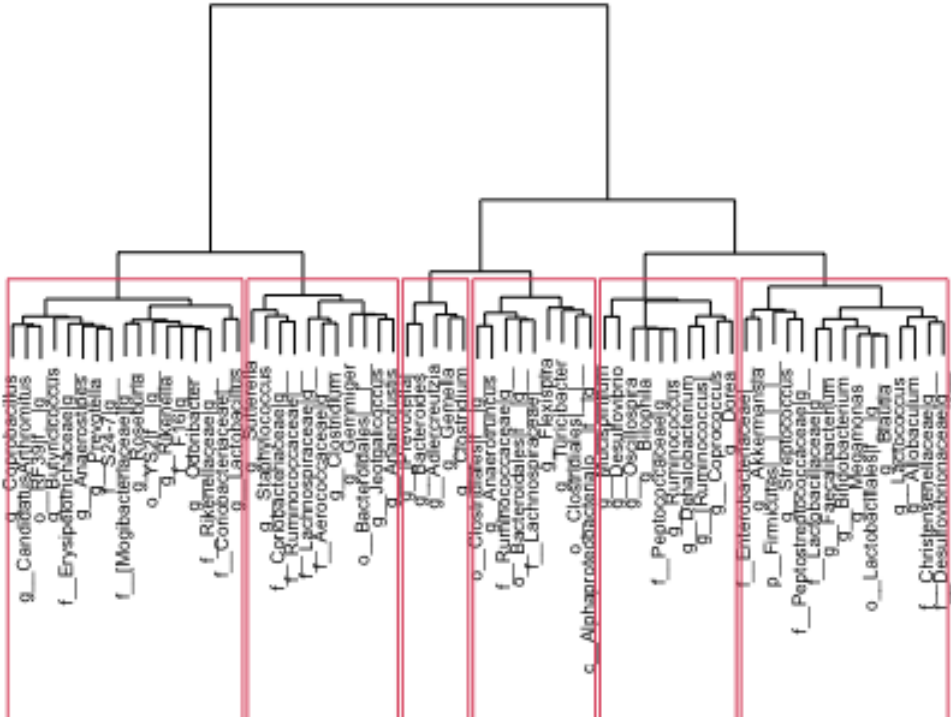

CAG 2

CAG 3

CAG 4

CAG 5

CAG 6

CAG 1

Supplement: Goto et al. supplementary material 1 — Goto et al. supplementary material [file S0007114525105916sup001.pdf]
