# Supplementary material for: Purified diet reduces intestinal IgA and alters the microbiota accordingly
Source: Br J Nutr. 2025 Dec 17;135(3):276–85. doi: 10.1017/S0007114525105916 (PMC12912838; doi:10.1017/S0007114525105916)
Supplement: Goto et al. supplementary material 2 — Goto et al. supplementary material [file S0007114525105916sup002.docx]

Supplemental table 1. Body weight gain, water intake, and feed intake of experimental animals.

| Parameter | PD | NPD |
| --- | --- | --- |
| Body weight gain (g) | 0.60 ± 0.38 | 0.90 ± 0.88 |
| Water intake (mL/day) | 2.13 ± 0.09 | 3.75 ± 0.56 |
| Feed intake (kcal/day) | 13.0 ± 2.57 | 12.7 ± 0.32 |

Data shown are expressed as mean ± SE. (**p* < 0.05, ^#^*p* < 0.1, Mann-Whitney U test.)
